# Pharmacological classes that extend lifespan of *Caenorhabditis elegans*

**DOI:** 10.3389/fgene.2015.00077

**Published:** 2015-03-03

**Authors:** Maria Carretero, Rafael L. Gomez-Amaro, Michael Petrascheck

**Affiliations:** ^1^Department of Chemical Physiology, The Scripps Research Institute, La Jolla, CA, USA; ^2^Department of Molecular and Experimental Medicine, The Scripps Research Institute, La Jolla, CA, USA; ^3^Department of Molecular and Cellular Neuroscience, The Scripps Research Institute, La Jolla, CA, USA

**Keywords:** aging, lifespan, pharmacology, GPCR, drug discovery, *Caenorhabditis elegans*, dietary restriction mimetics

## Abstract

Recent progress in the field of aging has resulted in ever increasing numbers of compounds that extend lifespan in *Caenorhabditis elegans*. Lifespan extending compounds include metabolites and synthetic compounds, as well as natural products. For many of these compounds, mammalian pharmacology is known, and for some the actual targets have been experimentally identified. In this review, we explore the data available in *C. elegans* to provide an overview of which pharmacological classes have potential for identification of further compounds that extend lifespan.

## INTRODUCTION

As a consequence of the seminal discoveries demonstrating that lifespan can be modulated by genes, it became clear that lifespan might also be extended using chemicals (Johnson, 1990; Kenyon et al., 1993). This concept has certainly been demonstrated, and today many compounds have been identified that extend lifespan in model organisms such as worms, flies and even mice (Kang et al., 2002; Howitz et al., 2003; Evason et al., 2005; Baur et al., 2006; Wilson et al., 2006; Petrascheck et al., 2007; Benedetti et al., 2008; McColl et al., 2008; Harrison et al., 2009; Pietsch et al., 2009; Alavez et al., 2011; Moskalev and Shaposhnikov, 2011; Oxenkrug et al., 2012; Martin-Montalvo et al., 2013; Priebe et al., 2013; Ye et al., 2014). Among all of these model organisms, *Caenorhabditis elegans* stands out because of the large variety of compounds known to extend lifespan. It is now possible to group these compounds into pharmacological classes, and use these groupings as starting points to search for additional lifespan extending compounds.

In the following paragraphs, we will give a short overview of what is known about the pharmacology of lifespan extension in *C. elegans*. We will summarize the discovery of lifespan extending compounds, comment on problems associated with the interpretation of pharmacological data, and we will end with a discussion of specific pharmacological classes of lifespan extending compounds.

## DISCOVERING LIFESPAN EXTENDING COMPOUNDS

There are two fundamentally different approaches to identify compounds that have a desired biological effect. These two approaches are often referred to as forward and reverse pharmacology, analogous to forward and reverse genetics (Bartfai, 2006). Forward pharmacology approaches, also called phenotypic screens, screen for compounds that elicit a desired phenotype, like the extension of lifespan. While forward pharmacology is intuitively appealing, as it searches for the desired effect, it has a number of drawbacks. The first is that screens must generally be conducted *in vivo*. *In vivo* screens are more complex, generally longer, and have higher costs associated than *in vitro* screens. Even if these disadvantages are overcome, elucidating the mechanisms by which a hit-compound achieves the desired effect is difficult. Elucidating drug mechanisms generally requires the identification of the drug target, which even today represents a major challenge (i.e., the binding target of the compound).

Reverse pharmacology circumvents the problem of target identification by screening for compounds that bind to, or inhibit, the function of a specific protein target. Reverse pharmacology screens are largely done *in vitro*, and offer the ability to screen very large chemical libraries (+500,000). Targets are validated based on prior knowledge, such as genetic studies in model organisms or gene association studies in humans affected by the disease. However, target validation, or choosing the protein target against which to develop a drug, also poses considerable difficulties (Pankevich et al., 2014).

As the process of aging is not easily replicated *in vitro*, most lifespan extending compounds have been identified by simply testing whether or not a given compound extends lifespan in a model organism (forward pharmacology). Thus far, most compounds that have been tested for their ability to extend lifespan had prior known pharmacology. Initially, these compounds were developed to inhibit a specific target, independent of their effect on aging. Only later were they tested for their ability to extend lifespan in *C. elegans* or other organisms. Thus, at its current state, the pharmacology of aging is a hybrid of forward and reverse pharmacology.

Why is the pharmacology of aging a hybrid of these two approaches? The simple answer is that it suffers from the disadvantages of both approaches. Target validation is problematic because most of the genes thus far found to be involved in the determination of lifespan are either essential, or they affect mitochondrial biology, insulin signaling, or general metabolism (Lee et al., 2003; Curran and Ruvkun, 2007; Hansen et al., 2007; Smith et al., 2008). These are difficult targets, as any lifespan extending compound will be given to old and frail people over extended periods of time, and therefore demands an extremely good safety profile. The history of the development of anti-obesity drugs has shown how difficult it is to choose an appropriate target to modulate metabolism in safe ways. While the example of metformin shows that it is possible to safely modulate insulin and/or general metabolism (Onken and Driscoll, 2010; Martin-Montalvo et al., 2013), we should note that the glucose lowering effects of metformin were discovered accidently through malaria research and not by a screen based on a validated target (Bailey and Day, 2004; Madiraju et al., 2014)

The forward pharmacology approach has logistical problems. Screens for lifespan in mammals are prohibitively expensive, and thus screens must be conducted in small model organisms. Even for *C. elegans*, maintaining, treating, and scoring thousands of cohorts of animals to determine their lifespan is inherently difficult, as even small irregularities have considerable effects on lifespan. For these reasons, few medium (>1000) to large (>10,000) screens have been performed using diverse compound libraries (Petrascheck et al., 2007; Ye et al., 2014). Lifespan data in and of themselves are problematic, as they are non-normally distributed, and Z’ statistics generally used to evaluate other screens are not appropriate (Zhang et al., 1999). Thus, unless some groundbreaking technical advances are made, the pharmacology of aging is likely to remain a hybrid of forward and reverse pharmacology for the foreseeable future.

Early studies in *C. elegans* used extremely high concentrations of chemicals, creating the impression that worms were especially resistant. However, today many of the lifespan extending compounds are effective at concentrations in the lower micromolar range (Luciani et al., 2011; Ye et al., 2014). When compared to cell culture, these concentrations still seem high, but compared to mouse studies they are not. Drug injections are generally conducted at concentrations of 5–200 mg/kg, resulting in an internal concentration in the lower micromolar range (Hayashi and McMahon, 2002). As concentrations for *C. elegans* are indicated for the external culture medium, the internal concentrations are likely to be lower and thus similar to those in mice.

Interpreting lifespan data obtained from compounds with known pharmacology has its own pitfalls. The pharmacological data available for most lifespan extending compounds are based on human data, while the lifespan data are based on experiments in model organisms (Knox et al., 2011). How well pharmacology between species is conserved is unknown, as we have no method to determine all protein targets of a compound. It may well be that a compound annotated as an inhibitor for a specific kinase extends lifespan by inhibiting an off-target.

Thus, after the identification of a lifespan extending compound, it is important to test multiple, structurally different compounds with the same pharmacology. If several structurally different compounds with the same pharmacology extend lifespan, the lifespan extending effect is likely to come from the annotated target, since off targets tend to be different for different structures. For example, multiple serotonergic antagonists extend *C. elegans* lifespan irrespective of their structure (Ye et al., 2014). Furthermore, combining structural studies with genetic studies, in which the compound is tested on mutants lacking the suspected target, allows the identification of the compound target with a high degree of certainty.

Given these caveats, we will discuss only pharmacological classes for which (i) multiple compounds were identified and (ii) additional genetic data exist that support the notion that targeting this class of proteins will lead to a lifespan extension. The genetic data we considered include mutations in target proteins that either cause a lifespan extension or, as is often the case, abrogate the lifespan extending effect of the compound. As an exception, we include natural compounds because of their general interest and wide use as food-supplements.

## PHARMACOLOGICAL CLASSES THAT EXTEND LIFESPAN

### ANTIOXIDANTS

Because of Harman’s theory of oxidative stress, antioxidants were some of the first compounds to be tested for their ability to extend lifespan and, as a compound class, have received quite a bit of attention (Harman, 1972; Melov et al., 2000; Benedetti et al., 2008). Indeed, antioxidants that extend *C. elegans* lifespan have been identified. These findings initially lent support to the idea that oxidative stress causes aging. However, later experiments guided by the theory of hormesis have challenged this view of aging. The theory of hormesis predicts that low levels of stress extend lifespan by over-activating stress responses, leading to excess stress response capacity and thus stress resistance and long-lived animals. Several reactive oxygen species (ROS) generating compounds such as rotenone, paraquat, arsenite, or naphthoquinone derivatives, were found to extend lifespan of *C. elegans* when used at low concentrations (Schulz et al., 2007; Van Raamsdonk and Hekimi, 2009; Lee et al., 2010; Hunt et al., 2011). Surprisingly, antioxidants blocked the lifespan extension of many hormetic agents, clearly suggesting that oxidative stress was required for the lifespan extension. Therefore, evidence exists that antioxidants extend lifespan and, paradoxically, also abrogate lifespan extension by hormetic agents. How can these findings be unified? One argument is that, although a compound has antioxidant capabilities, it still may extend lifespan by blocking an enzymatic activity independent of the antioxidant effect. This may indeed be true for some antioxidants. However, given the evidence that oxidative stress increases with age, it is unlikely to be true for all antioxidants. An interesting alternative that could explain how oxidants (hormetic agents) as well as antioxidants extend lifespan is to hypothesize that redox regulation plays an important role in determining lifespan. For example, many proteins known to be involved in aging (e.g., DNA repair enzymes) contain reactive cysteines whose redox status may alter protein activity (Weerapana et al., 2010).

While lifespan extending antioxidants were found based on candidate approaches, unbiased screens testing many pharmacological classes for their ability to extend *C. elegans* lifespan did not result in any lifespan extending antioxidants. This observation suggests that, as a pharmacological class, antioxidants may not be a particularly strong candidate for identification of lifespan extending compounds (Ye et al., 2014).

### METABOLITES

The first ever intervention found to verifiably extend lifespan was dietary restriction. Thus, dietary restriction immediately linked the process of aging to metabolism. In recent years, metabolites have received increased interest, due in part to technical advances in metabolomics and the identification of metabolic enzymes important in the determination of lifespan. Today, multiple metabolites are known that play a role in the determination of adult lifespan. These include the nuclear hormone receptor ligand dafachronic acid, ascarosides (ascr#2, ascr#3), *N*-acylethanolamines, and α-ketoglutarate (Motola et al., 2006; Lucanic et al., 2011; von Reuss et al., 2012; Ludewig et al., 2013; Priebe et al., 2013; Chin et al., 2014).

Ascarosides are endogenously produced compounds that are derived from the dideoxy sugar ascarylose, and were discovered as high population density signals. They affect many aspects of *C. elegans* physiology. For example, the lifespan extension of ascr#2 is mediated by the G-protein coupled receptor (GPCR) DAF-37, specifically expressed in the nervous system. This is an excellent example of how environmental signals are able to affect longevity via the nervous system (von Reuss et al., 2012).

Dafachronic acids are bile-acid-like steroids that are ligands for nuclear hormone receptors and are synthesized in starving animals. In adult *C. elegans*, dafachronic acids directly or indirectly activate NHR-8, leading to a reduction in *let-363*/mTOR expression and subsequent longevity (Motola et al., 2006; Thondamal et al., 2014).

Similarly, the tricarboxylic acid cycle intermediate α-ketoglutarate appears to mediate dietary restriction induced lifespan extension. Starvation induces α-ketoglutarate synthesis, which then blocks the ATP synthase, leading to reduced oxygen consumption, reduced ATP synthesis and increased autophagy (Chin et al., 2014).

In contrast, *N*-acylethanolamines, a class of fatty acid amides, are synthesized under favorable conditions. While high levels of *N*-acylethanolamines block lifespan extensions by dietary restriction, low levels of *N*-acylethanolamines are found under conditions of dietary restriction and are sufficient to extend lifespan (Lucanic et al., 2011). There are still few metabolites known to regulate lifespan, but the aforementioned examples show that the metabolome is likely to contain many more interesting compounds involved in the regulation of lifespan.

### KINASE INHIBITORS

The first cloned gene found to be important for lifespan determination was the class-I phosphatidylinositol 3-kinase *age-1* (Morris et al., 1996). In addition to *age-1*, numerous mutations in various kinases have been found to extend *C. elegans* lifespan, including the receptor tyrosine kinase *daf-2*, *akt-1*, TOR, and S6 kinase, to name a few. Mutations in kinases like *age-1* and the insulin/IGF receptor *daf-2* cause some of the most dramatic effects on lifespan. As mutations in kinases are also frequently found in cancers and other diseases (Vogt et al., 2010) many kinase inhibitors were found to extend *C. elegans* lifespan (e.g., lithium, GDC-0941, LFM-A13, and rapamycin), with the most promising being rapamycin (Harrison et al., 2009; Ye et al., 2014). However, thus far none of the tested kinase inhibitors has been able to reproduce the spectacular longevity seen in *age-1* or *daf-2* mutants. For example, PI3kinase inhibitors like GDC-0941 do increase *C. elegans* lifespan, but only by about 10%, while the most extreme *age-1* mutant alleles increase lifespan by 700% or more (Ayyadevara et al., 2008; Bharill et al., 2013). Similarly the IIS (*daf-2*) inhibitor NT219 blocks IIS signaling in *C. elegans*, but does not extend lifespan (El-Ami et al., 2014). It is unclear why these kinase inhibitors are unable to mimic the effect of genetic mutations. Possible explanations include: (i) the kinase inhibitors are not specific enough, and thus cause side effects masking longevity, (ii) inhibiting the kinase activity only is insufficient, (iii) longevity in the mutants is partially due to altered development. These three possibilities are testable and, while currently the answer is not known, it should be possible to clarify these issues soon.

### NUCLEAR HORMONE RECEPTORS

Nuclear hormone receptors are an important class of regulatory proteins that activate or repress gene expression patterns in response to cellular signals. The fact that these signals generally consist of small molecules, like steroid hormones, makes nuclear hormone receptors important drugs targets. Because of the early discovery of the role of DAF-12 in the determination of lifespan, hormonal regulation of lifespan has been a central theme in aging. Candidate approaches, as well as unbiased screens of synthetic compounds have identified lifespan extending compounds known to bind nuclear hormone receptors. One problem with studying nuclear hormone receptors using *C. elegans* is its vastly expanded repertoire of 284 nuclear hormone receptors, compared to 49 in mammals (Antebi et al., 2000; Robinson-Rechavi et al., 2001; Motola et al., 2006) making it difficult to translate *C.elegans* findings to mammals.

### G PROTEIN COUPLED RECEPTOR LIGANDS

Compounds affecting GPCR are among the most important pharmacological classes for drug discovery (Paolini et al., 2006; Yildirim et al., 2007). In medium scale screens for compounds with known pharmacology that extend lifespan, 50% of all hit compounds targeted GPCRs (Ye et al., 2014). This was surprising, as the genome wide RNAi screens only uncovered one GPCR as being involved in the regulation of lifespan (Lee et al., 2003; Hansen et al., 2007). How can these findings be unified? One explanation may be a technical difference. The RNAi screens started to inhibit gene expression early in development, while the chemical screens started inhibition during adulthood. Indeed, the lifespan extending effect of Mianserin was reduced from +33% to less than 10% when added to developing animals rather than adult animals (Petrascheck et al., 2007). This result suggests that GPCRs signal environmental changes, upon which the physiology of the animal adapts. Thus, blocking signaling in adults causes a change that leads to alterations in physiology, while a continuous repression starting in early development does not. While considered “accidental” pharmacology, finding bioactive compounds by chance often results in compounds targeting GPCRs. Examples include Cannabinoid receptors, Opioid receptors, and dopamine receptors to name a few. By contrast, forward genetic screens, which result in the permanent inhibition of a GPCR, have only identified a handful of GPCRs. Out of roughly 6000 *C. elegans* genes cloned, only five were GPCRs, despite the fact that GPCRs represent 5% of the entire genome (Moresco and Koelle, 2004). Thus, it appears that GPCRs exist that must be active during development in order to affect lifespan when blocked in adults, probably because their function is to modulate lifespan in response to environmental change.

### NATURAL COMPOUNDS

Many natural compounds or plant extracts such as blueberry polyphenols, curcumin, quercetin, ginkgo extracts, and resveratrol have been identified to extend *C. elegans* lifespan (Wu et al., 2002; Howitz et al., 2003; Wilson et al., 2006; Pietsch et al., 2009). What makes a natural compound approach attractive is that plant extracts are generally regarded as safe, and are often used as food supplements. However, natural compounds are hard to synthesize and modify, and thus target identification is particularly difficult for natural compounds. The ongoing dispute on the mechanism of action of resveratrol certainly gives testimony on such difficulties. The proposed mechanisms range from antioxidant properties, to activation of the histone deacetylase SIRT1, AMPK activation by blocking phosphodiesterase activity, or mimicking tyrosine-activating stress response pathways via TyrRS and PARP1 (Howitz et al., 2003; Park et al., 2012; Sajish and Schimmel, 2014).

## SUMMARY

In this short review, we have discussed how lifespan extending compounds are currently identified, what limits progress, and which pharmacological actions seem to extend lifespan. Discussing the different classes above shows that lifespan pharmacology is not inherently different from any other type of drug discovery. It seems that the same pharmacological classes (GPCR’s, nuclear hormone receptors, Kinases) that delivered compounds for many other indications are also suitable for lifespan extension. It may, however, be that our conclusions are biased for historical reasons. Most of the compounds tested for lifespan were those that were readily available, which are the same compounds that stand out as promising for other indications. Alternatively, it is also possible that GPCRs, nuclear hormone receptors, kinases, and ion-channels make better drug targets. This may be because all these proteins exert regulatory functions, affect multiple physiological functions, and may also be easier to target with drugs than others. Whatever the reasons, the large overlap between pharmacological classes that extend *C. elegans* lifespan and drugs used in humans is encouraging because it suggests that some degree of conservation can be expected.

### Conflict of Interest Statement

The authors declare that the research was conducted in the absence of any commercial or financial relationships that could be construed as a potential conflict of interest.
